# Viscoelastic Hemostatic Assays for Postpartum Hemorrhage

**DOI:** 10.3390/jcm10173946

**Published:** 2021-08-31

**Authors:** Alyson E. Liew-Spilger, Nikki R. Sorg, Toby J. Brenner, Jack H. Langford, Margaret Berquist, Natalie M. Mark, Spencer H. Moore, Julie Mark, Sara Baumgartner, Mary P. Abernathy

**Affiliations:** 1 Department of Obstetrics and Gynecology, University of Oklahoma College of Medicine, Oklahoma City, OK 73104, USA; alyliewspilger@gmail.com; 2 Indiana University School of Medicine South Bend Campus, Notre Dame, IN 46617, USA; nikrsorg@iu.edu (N.R.S.); natmark@iu.edu (N.M.M.); jpmark1959@gmail.com (J.M.); 3 Division of Natural Sciences, Indiana Wesleyan University, Marion, IN 46953, USA; toby.brenner@myemail.indwes.edu; 4 College of Pharmacy and Health Sciences, Butler University, Indianapolis, IN 46208, USA; jlangford@butler.edu; 5 College of Science, University of Notre Dame, Notre Dame, IN 46556, USA; mberquis@nd.edu; 6 Marian University College of Osteopathic Medicine, Indianapolis, IN 46222, USA; spencerhamiltonmoore@gmail.com; 7 Department of Obstetrics and Gynecology, Saint Joseph Regional Medical Center, Mishawaka, IN 46545, USA; 8 Department of Obstetrics and Gynecology, Indiana University School of Medicine, Indianapolis, IN 46202, USA; mabernat@lightbound.com

**Keywords:** thromboelastography, rotational thromboelastometry, postpartum hemorrhage, pregnancy, blood component transfusion, blood coagulation, obstetrics, disseminated intravascular coagulation, amniotic fluid embolism, von Willebrand factor

## Abstract

This article discusses the importance and effectiveness of viscoelastic hemostatic assays (VHAs) in assessing hemostatic competence and guiding blood component therapy (BCT) in patients with postpartum hemorrhage (PPH). In recent years, VHAs such as thromboelastography and rotational thromboelastometry have increasingly been used to guide BCT, hemostatic adjunctive therapy and prohemostatic agents in PPH. The three pillars of identifying hemostatic competence include clinical observation, common coagulation tests, and VHAs. VHAs are advantageous because they assess the cumulative contribution of all components of the blood throughout the entire formation of a clot, have fast turnaround times, and are point-of-care tests that can be followed serially. Despite these advantages, VHAs are underused due to poor understanding of correct technique and result interpretation, a paucity of widespread standardization, and a lack of large clinical trials. These VHAs can also be used in cases of uterine atony, preeclampsia, acute fatty liver of pregnancy, amniotic fluid embolism, placental abruption, genital tract trauma, surgical trauma, and inherited and prepartum acquired coagulopathies. There exists an immediate need for a point-of-care test that can equip obstetricians with rapid results on developing coagulopathic states. The use of VHAs in predicting and treating PPH, although in an incipient state, can fulfill this need.

## 1. Introduction

Postpartum hemorrhage (PPH) is the leading cause of maternal morbidity and mortality throughout the world [1,2,3]. Its prevalence has risen around the globe despite advancements in obstetric protocols for preventing and treating massive hemorrhage [4,5]. Management protocols for diagnosing and treating PPH are characterized by heterogeneous and conflicting definitions and classifications [6,7]. For trauma, the classification of severity and definition of severe bleeding and resuscitation strategies are more straightforward, whereas the definition of PPH is more ambiguous [8]. For example, many define PPH as blood loss >500 mL after vaginal delivery or >1000 mL after cesarean delivery [3,6,9]. The American College of Obstetricians and Gynecologists formerly used this definition but have updated their most recent PPH Practice Bulletin to define PPH as either cumulative blood loss ≥1000 mL or blood loss accompanied by the signs and symptoms of hypovolemia, regardless of delivery route [10]. The Royal College of Obstetricians and Gynecologists classifies PPHs into categories of minor (500–1000 mL blood loss) and major (>1000 mL blood loss) [11]. Others define PPH as a fall in hemoglobin by ≥4 g/dL, the need for transfusion of ≥4 units, or the requirement of massive transfusion [12,13]. Some consider secondary PPH to be a clinical diagnosis defined by excessive bleeding between 24 h and up to 12 weeks after delivery [10,11,14]. Another major limitation for predicting and treating PPH is that the amount of blood lost is difficult to assess and is often underestimated regardless of whether analysis is done clinically or with objective tools [13,15]. Using markers of hemostatic competence as diagnostic adjuncts therefore has been proposed as a method to enhance the accuracy of classifying PPH [8].

A multidisciplinary consensus statement recently provided guidelines for patient blood management for clinical practitioners managing perinatal care. The statement recommends using common coagulation tests (CCTs) or viscoelastic hemostatic assays (VHAs) to guide goal-directed blood component therapy (BCT), hemostatic adjunctive therapy (HAT), and prohemostatic agents in PPH. CCTs include platelet count, prothrombin time (PT), activated partial thromboplastin time (aPTT), and fibrinogen level; VHAs include thromboelastography (TEG) and rotational thromboelastometry (ROTEM) [8]. In recent years, TEG and ROTEM have increasingly been used to guide BCT and prohemostatic agents in PPH [16,17]. The novel approaches for the use of VHAs in PPH derive from the strategies and guidelines commonly used in cardiothoracic surgery and trauma resuscitation [3,18].

Assessment of hemostatic competence in PPH has three pillars: clinical observation, CCTs, and VHAs. There is no high-level data that suggests that any one method is better than the other [8,11,12,16]. Clinical observation, the first pillar of identifying PPH, includes assessment of vital signs, quantification of blood loss, and identification of the source of bleeding via physical examination, at which time appropriate treatment and intervention may be initiated [10]. Clinical observation alone is potentially insufficient in that it creates variation of the definition of PPH and the diversity of the time period over which PPH evolves [6,7]. The second pillar involves using the CCTs to monitor hemostasis postpartum. The main advantage of this pillar is that CCTs have wide availability and a high level of quality control and reproducibility [16,18]. However, laboratory tests with lengthy turnaround times may be limited in the context of rapid hemorrhage and will not reflect current hemostatic competence of the patient [8,11,19,20,21,22,23]. It has been well documented that PT and aPTT have low sensitivities for determining the existence of hypocoagulopathy [19,24]. Fibrinogen levels determined by the Clauss method can show hypocoagulability sooner than PT and aPTT can, and ought to be used to supplement CCT monitoring of PPH [8,11,19,25]. The final pillar uses VHAs to assess hemostatic competence. VHAs analyze the viscoelastic properties of whole blood and charts the entire process of clot formation from clot initiation through termination to fibrinolysis. In this way, VHAs have an advantage over CCTs in that they assess the cumulative contribution of all components of the blood throughout the entire formation of a clot [23]. VHAs also have faster turnaround times compared to CCTs and their correct application has been shown to decrease blood loss and blood product use in the postpartum period [8,11,23,26,27]. Expeditious turnaround time is vital to improve patient outcomes, particularly in patients with amniotic fluid embolism (AFE) who are at risk for an abrupt hemodynamic collapse [28,29]. Furthermore, since VHAs are point-of-care (POC) tests, they can be followed serially at the patient’s bedside in labor and delivery room, operating room, and in the emergency department for precipitous emergency cesarian section such as abruptio placentae [30]. Although these tests have clear value for monitoring hemostasis, VHAs are chronically underused because of a local variability of pipetting technique in non-cartridge forms of TEG and ROTEM, standardization of parameters as well as a lack of large clinical trials demonstrating benefit [31,32,33].

## 2. Historical Challenges in the Application of VHAs for PPH

Strategies for the use of VHAs to diagnose and treat PPH are derived from the literature that describes the use of VHAs in liver transplantation, cardiac surgery, and trauma. Important sentinel publications awaited long periods of gradual acceptance such that it was only in 1985 for liver transplantation and in 1999 for cardiac surgery that the first significant studies were completed demonstrating the advantages of POC VHAs in diagnosing coagulopathy and goal-directing BCT/HAT and prohemostatic therapy necessary for the prevention and treatment of hemorrhage in liver transplantation and heart surgery. Traumatologists waited from the first studies of 1997 until April 2012 when the total number of patients whose trauma resuscitation was guided by VHAs surpassed 1600. Five months later in September 2012, a single-center, retrospective study of 1974 patients confirmed the benefit of VHA-guided resuscitation for hemorrhaging trauma patients [34,35,36,37,38,39,40,41,42,43,44,45,46,47]. The use of VHAs in obstetrics has grown much slower compared to liver transplantation, cardiac surgery, and trauma. This delay may in part be due to the significant complexity of the definition of PPH. Additionally, the adoption of VHAs in the PPH treatment may also be contributed to the lack of established transfusion trigger values, which have only been published recently [48,49,50]. A review of the timeline for the adoption of VHAs in trauma revealed a significant lag of approximately a decade which has been reproduced for PPH [3,12,25,51,52,53,54,55,56,57,58,59,60]. This across the board delay in the use of VHAs to guide BCT and HAT has been commented on in the literature [61]. However, the meteoric use of VHAs in the first year of the COVID-19 pandemic has been reported in 1417 patients as of April 2021 [62]. This rapid acceptance by the many specialists who care for COVID-19 patients reflects the decades long history of successful resuscitation in liver transplantation, cardiac surgery, and trauma. One can anticipate that the future of VHA-guided resuscitation for PPH will undergo a more rapid acceptance because of the expansion of VHA use into critical care during the pandemic [63,64,65,66].

In 2013, the incidence rate of PPH was reported to be just under three percent in the United States [4]. PPH largely presents without any predictive signs and often in the absence of any predisposing conditions, therefore, all women are considered to be at risk for PPH [67,68]. Because bleeding during and after childbirth is expected, healthcare practitioners are liable to overlook early signs of serious hemorrhage, endangering patients who will then be more likely to bleed for an extended period [6,7,16]. Clinical observation alone is insufficient to keep abreast of such a rapidly evolving situation, which can result from a myriad of complications. For instance, the AFE-associated coagulopathy is diagnosed and treated in a totally distinct fashion than the less sudden coagulopathy associated with uterine atony, the most typical cause of severe PPH [68,69,70].

The literature has described reference ranges for TEG and ROTEM parameters that have been endorsed by many institutions and obstetricians. Nonetheless, no internationally accepted values exist to allow uniform definition of triggers for diagnosing severe PPH or for standardizing BCT/HAT for patients with PPH. Headway is being made through the establishment of standards on the local level. Large studies have shown that VHA-guided goal-directed therapy results in better patient outcomes, specifically in patients with severe bleeding [28,60]. Currently, the optimal strategy for identifying and treating PPH employs clinical observation, empiric blood management, CCTs with Clauss fibrinogen, and VHAs [8,11,12,16]. The use of VHAs in predicting and treating PPH is in an incipient state similar to that of their use in trauma resuscitation more than a decade ago.

Instructions for the identification and treatment of PPH using TEG and ROTEM parameters differ considerably because of the paucity and heterogeneity among published studies regarding the use of VHAs to anticipate PPH and guide therapy. The three pillars of identifying PPH—clinical observation, CCTs, and VHAs—should be used concurrently to identify and treat PPH because its coagulopathy is complex and cannot be investigated through use of the CCTs alone. Studies have established a correlation between low fibrinogen levels and the incidence of PPH, but the low sensitivity and long turnaround time of this CCT make it insufficient for guiding BCT/HAT in PPH patients [71,72]. There is evidence in the utility of ROTEM in PPH as demonstrated by correlation between FIBTEM A5 and classical Clauss fibrinogen levels [48,73].

## 3. The Use of VHAs to Guide BCT and Hemostatic Adjunct Therapy in PPH

The use of TEG and ROTEM in the identification of hypocoagulopathy and guiding BCT/HAT in PPH is rising in popularity. Only recently have TEG and ROTEM been increasingly studied within the context of PPH [2,3,21,25,52,53,54,55,58,59,74,75,76,77,78,79,80,81,82,83,84,85,86,87,88,89,90,91,92]. BCT/HAT can be tailored to this patient population’s specific needs using TEG or ROTEM parameters.

**Figure 1:**
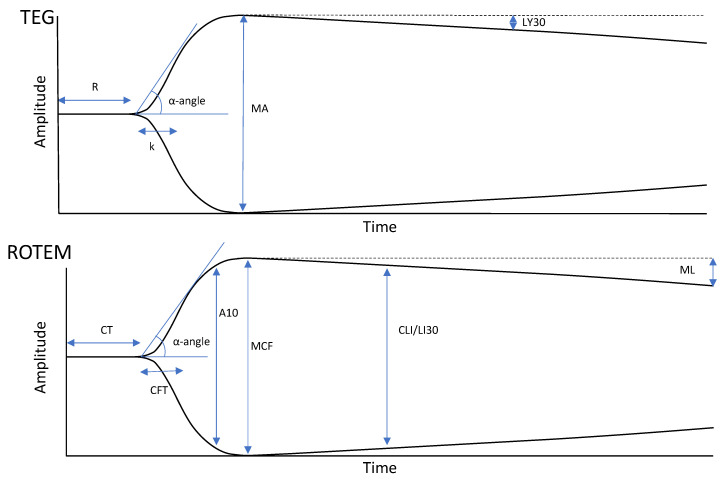
Depictions of normal or physiologic TEG (top) and ROTEM (bottom) tracings. TEG and ROTEM use equivalent but independently labelled parameters. Reaction time (R) and clot time (CT) refer to the time required for the transducer to be displaced 2 mm, correlating with the parameters of the PT/aPTT tests. Clot formation/kinetics (k) and clot formation time (CFT) are measures of initial clot strength and clot formation kinetics, referring to the time required for the transducer to be displaced 20 mm after it reached the 2 mm mark. α-angle is the angle formed between the horizontal axis and the sloped line formed between 0 and 20 mm of amplitude and is used in both TEG and ROTEM technologies. CFT/k and α-angle are broadly correlated with fibrinogen levels. Clot amplitude at 5/10 min (A5/A10) are measurements of the amplitude at 5-min intervals after the CT. Maximum amplitude (MA) and maximum clot firmness (MCF) refer to the maximum displacement acquired and are measures of maximum clot strength. They correlate with maximum clot retraction as a reflection of the crosslinking of fibrin with platelets. TEG and ROTEM analyzers also use differing parameters to describe fibrinolysis. Lysis at 30 min (LY30) shows the percent decrease in amplitude 30 min after achieving MA. Clot lysis index at 30 min (CLI30) is the residual clot remaining 30 min after CT, measured as a percentage of MCF. Maximum lysis (ML) is a measure of the percent decrease in amplitude at the end of the run. Adapted from [23] with permission from Semin Thromb Hemost., 2020.

In its most basic terms, the TEG and ROTEM curves represent the four stages of the lifespan of a clot from initiation, amplification, propagation, and termination through fibrinolysis. Figure 1 offers a summary explaining the correlation between each parameter as a reflection of the hemostatic competence of each phase of coagulation from the plasmatic phase through fibrin cross linking, to fibrin-platelet contraction, and finally clot lysis.

A prolongation in clot initiation, represented by the reaction time (R) in TEG and the clotting time (CT) in ROTEM, suggests a coagulation factor deficiency and warrants treatment with fresh frozen plasma (FFP), prothrombin complex concentrate, or specific factor replacement when required. The speed of clot development, represented by the kinetics time (k) and α-angle in TEG and the clot formation time (CFT) and α angle in ROTEM, historically represents the speed of fibrin formation; cryoprecipitate or fibrinogen concentrate is given for a prolongation in k/CFT or decrease in α-angle. A decrease in the maximum amplitude (MA) in TEG or maximal clot firmness (MCF) in ROTEM represents a decrease in overall clot strength, which can be attributed to either a platelet or fibrinogen pathology and can be treated with either platelets, cryoprecipitate, or fibrinogen concentrate. The A5/A10 are ROTEM parameters that measure the amplitude of the tracing at 5/10 min after the end of CT. The standard TEG and ROTEM tracings can also be compared to standard TEG and fibrinogen-specific tracings: TEG functional fibrinogen and fibrinogen thromboelastometry (FIBTEM). These eliminate the contribution of platelets to better distinguish the cause of the reduced clot strength and to treat the patient accordingly. These tests are used to detect fibrinogen deficiency and to guide fibrinogen supplementation. Excessive clot lysis, represented by lysis at 30 min (LY30) in TEG and the CLI30 and the maximal lysis (ML) in ROTEM, indicates hyperfibrinolysis and the potential need for tranexamic acid (TXA) (Figure 1) [3,19,20,56,73,93,94,95,96]. Emerging VHAs, such as ClotPro and Quantra, provide more advanced assessments of clot dynamics; however, their use in obstetrics has yet to be documented, and thus, have not been included in this review.

In surgical patients who have postoperative bleeding, a normal VHA tracing indicates an anatomic cause of bleed which requires surgical intervention since the patients do not have an underlying coagulopathy [97]. Much like in surgery, patients who have an anatomic cause of bleeding such as uterine atony, retained placenta, or genital tract trauma have a normal PT and aPTT and most commonly have a fibrinogen level >200 mg/dL, but these laboratory tests have lengthy turnaround times and will not demonstrate hemostatic competence of the patient until much later [16,17]. VHAs will show a normal tracing early in cases of atony, retained placenta, and trauma. The rapid turnaround time of this information allows clinicians to escalate obstetric care without administering fibrinogen concentrate or cryoprecipitate unnecessarily [8,11,17,19,20,21,22,23,26,27].

### 3.1. FFP, Cryoprecipitate, or Fibrinogen Concentrate

In PPH, fibrinogen is the first coagulation factor to diminish. Administration of sufficient fibrinogen to return levels to normal is needed to enable a recovery to hemostatic competence in severe PPH [52,55,71,98,99,100]. It has recently been recommended that Clauss fibrinogen levels of around 300–400 mg/dL and a FIBTEM of >16 mm are required to reverse the hypocoagulopathy and maintain hemostatic competence in a patient presenting with PPH. When these parameters are met, no further blood components are likely needed to be administered [28,98]. Preemptive treatment of fibrinogen to reduce the need for blood product transfusions in patients with PPH has been studied, specifically by Wikkelso et al. in 2015. In this randomized controlled trial of 249 patients, 2 g of fibrinogen concentrate were used as a preemptive treatment for patients who were initially normofibrinogenaemic and later developed PPH. No significant decrease in the administration of blood products for patients that experienced PPH was observe. Further studies have been proposed in order to determine the value of VHAs to diagnose and treat the coagulopathy due to PPH [101].

Regarding fibrinogen deficiency, there are three options available for correcting these deficiencies: FFP, cryoprecipitate, and fibrinogen concentrate. FFP has traditionally been the treatment used to replenish fibrinogen in PPH. Recently, studies have advocated for the early administration of fibrinogen concentrate or cryoprecipitate instead of FFP because FFP contains lower concentrations of fibrinogen than the plasma of a woman at term. FFP transfusion would then cause further hemodilution of fibrinogen [72,98,102,103,104]. While FFP use may cause volume overload, the use of cryoprecipitate or fibrinogen concentrate does not. European studies have found that fibrinogen concentrate use resulted in a reduction of blood product use, particularly when using a VHA-guided algorithm [104,105,106].

POC testing via VHAs enables obstetricians to focus both on volume of red blood cells and FFP for resuscitation and assess the levels of fibrinogen in PPH patients. For patients whose fibrinogen levels have risen to over 200 mg/dL and whose extrinsic thromboelastometry (EXTEM), a specialized form of ROTEM that focuses on the extrinsic pathway, clotting time is prolonged, FFP administration has been recommended to replenish volume [11,17,57]. This strategy relies on an understanding of how critical fibrinogen levels are in patients with PPH. Low fibrinogen level is an integral part of PPH coagulopathy as compared to that of TIC [71,107,108]. This strategy also supports the EXTEM A5 and FIBTEM A5 as the two primary ROTEM parameters to be analyzed in patients with PPH.

### 3.2. Prothrombin Complex Concentrate

VHAs have been used to guide prothrombin complex concentrate for hemostatic resuscitation [73,109]. It has been recently suggested that the administration of the prohemostatic agents Factor VIIa and desmopressin can be guided by VHAs in PPH patients [73,109,110]. The discordant nature of guidance regarding the administration of prohemostatic therapy in patients with PPH is a function of the emergent state of the use of VHAs in peripartum management. The future of BCT/HAT and prohemostatic therapy in patients with PPH may lie in application of precision-based medicine in which therapy is dictated by hemostatic phenotypes and guided by VHAs [102,111,112,113].

### 3.3. Platelets

Platelet function in patients suffering from PPH has not been well described in the literature. CCTs measure platelet counts but provide no information on platelet function [102]. Standard VHAs are limited in their ability to detect platelet dysfunction; however, specialized assays have been developed to be used in conjunction with standard VHAs to provide this information [3]. The TEG Platelet Mapping (TEG-PM) and other similar POC platelet function tests could enhance diagnoses, but their utilization in PPH has been limited [102]. Additionally, non-VET point-of-care platelet function tests such as Multiplate and VerifyNow P2Y12 can be used to detect platelet dysfunction [114,115,116]. A recent study on peripartum management has proposed that the EXTEM A10, which exhibits the extrinsic pathway, and the FIBTEM A10, which exhibits the extrinsic pathway without the contribution of platelets, can monitor platelet function. The difference between the EXTEM A10 and FIBTEM A10 has been proposed as a parameter called PLTEM which is related to platelet activity [77].

### 3.4. Tranexamic Acid

Since the 2010 Clinical Randomization of an Antifibrinolytic in Severe Hemorrhage 2 (CRASH-2) Trial and the 2017 World Maternal Antifibrinolytic (WOMAN) Trial, the use of prohemostatic therapy has been of increasing relevance. These RCTs found improved mortality when TXA was administered within 3 h of the start of bleeding due to trauma and PPH [117,118]. Ensuing publications have identified variance in these conclusions, and for this reason, these trials have become a source of controversy [5,8,11,15,111,119,120,121,122,123,124,125,126,127,128,129,130,131,132]. However, it has also been suggested that fibrinolytic parameters of VHAs are not reliable for guiding the administration of TXA in PPH patients [73,109,110]. Therefore, more research needs to be done to determine if VHAs can be utilized to guide the administration of TXA in the context of PPH. It is important to note that when aprotinin thromboelastometry (APTEM), a specialized ROTEM assay which focuses on the effect of fibrinolytic enzymes, is used in conjunction with EXTEM, a normal MCF displayed on the APTEM when compared to an abnormal MCF on the EXTEM is indicative of hyperfibrinolysis and may warrant administration of TXA [24,73].

## 4. Specific Pathological Entities in PPH

### 4.1. Uterine Atony

Pathological hemostasis predisposes women to PPH and therefore utilization of VHAs to define hemostatic derangement may prevent unanticipated bleeding [16,133]. Physiologic postpartum hemostasis of placental blood flow depends greatly upon adequate myometrial contraction and mechanical compression of the spiral arteries. Uterine atony is the failure of this physiologic myometrial contraction in the immediate postpartum period and is the most common cause of PPH [2,3,9,15,25,70,74,134,135]. Risk factors for uterine atony include multiparity, multiple gestation, history of PPH, prolonged labor, and placenta previa [70,102]. When PPH is caused by uterine atony, it is common for women to not demonstrate early coagulopathy [16,17]. Because of this, FFP should not be administered before the hemostatic competence of the patient is assessed because hemostatic impairment is improbable [16].

### 4.2. Preeclampsia

Hypertensive disorders in pregnancy are a leading cause of maternal morbidity and mortality worldwide, second only to hemorrhagic causes [1]. Preeclampsia and related hypertensive disorders (e.g., hemolysis, elevated liver enzymes and low platelets (HELLP) syndrome, eclampsia, etc.) cause hemostatic aberrancies which most often manifest as hypercoagulability [136]. Although the pathophysiology of preeclampsia is not agreed upon and may involve a genetic predisposition to thrombophilia [137], evidence suggests the etiology relates to abnormal placentation, lack of spiral artery remodeling, and subsequent uteroplacental insufficiency and trophoblast ischemia [138]. The local apoptosis releases pro-inflammatory cytokines into the maternal circulation, provoking a systemic intravascular inflammatory response and endothelial dysfunction [138]. As a result, preeclamptic mothers procure an immunothrombotic state characterized by increased thromboxane A2, increased platelet consumption, and increased acute phase reactants (e.g., thrombin) [138,139]. Microthrombosis is routinely observed histologically in placentas and kidney biopsies of preeclamptic mothers, which likely explains the end-organ damage observed in this group [139].

In a retrospective Norwegian population study of 315,085 singleton births between 1999 and 2004, there was a significantly greater incidence of moderate (>500 mL) and excessive (>1500 mL) PPH in mothers who developed preeclampsia or HELLP syndrome [140]. The thrombohemorrhagic manifestations of these disorders require better diagnostic and therapeutic methods to prevent maternal and fetal complications. TEG offers the theragnostic potential to diagnose underlying coagulopathies and simultaneously guide blood products to provide safer peripartum care. Lidan et al. [141] demonstrated that, compared to CCTs, TEG provided more accurate information on the coagulation status of preeclamptic patients. Moreover, TEG values significantly differed for mothers with mild versus severe preeclampsia, which has implications for earlier prediction of disease severity [141].

Assessment of fibrinolysis is another advantage of VHAs over CCTs. A higher rate of fibrinolysis has been demonstrated in preeclamptic women compared to healthy pregnant women [142,143]. In studies specifically with preeclamptic women, it has been suggested that preemptive use of fibrinogen concentrate guided by FIBTEM has reduced the need for blood components and lowered the risk of circulatory overload, which can cause serious harm to women with preeclampsia although no RCTs have been performed [28,55].

### 4.3. Acute Fatty Liver of Pregnancy

Decreased oxidation of fatty acid chains leads to increased concentration of fatty acids in maternal serum, potentially leading to acute fatty liver of pregnancy (AFLP). AFLP can lead to maternal coagulopathy as well as electrolyte abnormalities [144]. AFLP may cause renal impairment which has exhibited severe changes in coagulopathy [145]. AFLP can be challenging to assess as it does not resolve prior to delivery. Katz et al. mentions an unexpected discrepancy between a guideline international normalized ratio (INR) and two cases of AFLPs’ INRs. AFLP INRs in two women were reported higher than the accepted guideline INR [110]. Even so, the prognosis has been improved by advances in critical care and urgent delivery. In treating the pathology, avoiding drugs that are potentially toxic to the liver and maintaining hepatic blood flow are paramount. FFP, platelets, and vitamin K are recommended treatments for coagulopathy caused by AFLP and the use of VHAs may improve prognosis [146]. VHAs can be used to monitor hemostatic competence, manage coagulopathy, and reduce blood product waste in women with AFLP [147].

### 4.4. Amniotic Fluid Embolism

AFE, a rare disorder in which amniotic fluid or debris enters the mother’s pulmonary circulation and causes an immunothrombotic response, may occur in healthy pregnant women in the second trimester of pregnancy, during cesarean section, or during or after vaginal delivery; additionally, it can occur during or after abortion or after abdominal trauma [148]. AFE reactions can be classified as anaphylactoid reactions or complement activations to fetal antigens or idiosyncratic reactions, which are caused by a combination of immunologic and vasospastic factors [149]. In AFE, disruption between maternal and fetal compartments causes consumption-coagulopathy, potentially leading to thrombotic obstruction of small and midsize vessels, contributing to organ dysfunction. Simultaneously, this consumption of platelets and coagulation proteins results in thrombocytopenia and low concentrations of clotting factors, which may cause hemorrhagic complications [150]. Observations in a case of AFE with sequential monitoring of blood samples suggest that fibrinolysis may precede coagulopathy [151]. AFE has been shown to result from hyperfibrinolysis, or complete clot lysis, in EXTEM but not APTEM tests [74]. AFE requires expeditious diagnosis and aggressive management due to its high associated mortality. Because of this, CCTs are impractical in monitoring evolving hemostatic competence and informing clinical decisions. POC testing via TEG and ROTEM can be used for more timely administration of targeted blood products [152].

Some 80% of patients with AFE will develop disseminated intravascular coagulation (DIC), a life-threatening condition with a wide range of clinical manifestations which results in generation of microvascular thrombi, leading to multiple organ dysfunction and, occasionally, severe bleeding [150,153,154]. DIC can be caused by an extensive range of clinical events, including AFE, severe preeclampsia, eclampsia, HELLP syndrome, and AFLP [155]. In DIC, all anticoagulant pathways are functionally impaired. The mononuclear cells of the vascular endothelium express tissue factor, resulting in thrombin generation and subsequent fibrinogen to fibrin conversion, contributing to microvascular clot formation. As DIC is always secondary to an underlying condition, the most important component of treatment of coagulopathy involves the diagnosis and management of the underlying condition [150].

**Figure 2:**
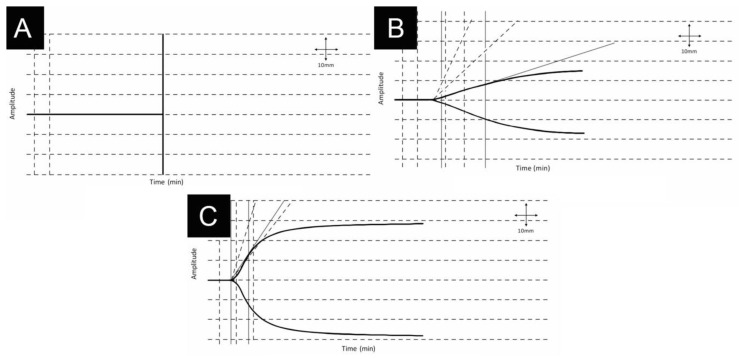
35-year-old woman presenting with a cardiac arrest due to an amniotic fluid embolism during delivery. Patient required cardiopulmonary resuscitation and developed immediate disseminated intravascular coagulation, respiratory and renal failure which required ventilation and eventual dialysis. The fetus was successfully delivered by immediate cesarean section and the patient completely recovered. First thromboelastography tracing reveals no clot formation (**A**). Improvement over 2 (**B**) and 8 (**C**) hours were achieved by following thromboelastography-guided massive transfusion and use of HAT. Physiologic thromboelastography tracing is also depicted (**C**) Adapted from [29] permission from *J. Extra Corpor. Technol.*, 2016.

For example, Figure 2 describes a patient with AFE who presented with a cardiac arrest and required TEG guided massive transfusion with HAT [29].

Much as occurred in trauma, there is a now growing demand for a POC test that can equip obstetricians with rapid results on developing coagulopathic states [2,3,8,12,16,17,19].

### 4.5. Placental Abruption

Placental abruption is associated with significant incidence of PPH and a greater percentage of patients with placental abruption will progress to significant PPH. Recent studies have shown that FIBTEM A5, EXTEM A5, and EXTEM CT can predict estimated blood loss greater than 2000 mL. TEG has been used to guide BCT/HAT for abruptio placenta since 1997 [51,56,76]. Women with this pathophysiology have a significantly prolonged EXTEM CT and lower FIBTEM A5 compared to those with PPH from other causes. These women also have a more severe coagulopathy than women with PPH from other causes and require higher fibrinogen concentrate doses to resolve the coagulopathy. The use of ROTEM for these patients individualizes treatment and allows for selective use of fibrinogen concentrate which both improved clinical outcomes and decreased blood product use [76].

### 4.6. Genital Tract Trauma and Surgical Trauma

Genital tract trauma and surgical trauma are common causes of significant PPH. The most recent studies have demonstrated a great decrease in the use of FFP, a reduction of the markers of severe maternal mortality, an elimination of the incidence of transfusion-associated circulatory overload (TACO), as well as a decrease in ICU admissions for those patients with PPH whose BCT/HAT is guided by ROTEM [76,81].

Genital tract trauma occurs in about 10% of PPH cases and is associated with instrumental delivery where the cervix or vagina is lacerated, or the uterus is distressed by delivery. After such a delivery, the obstetrician examines the genital tract and if there is evidence of trauma, or bleeding is demonstrated, ideally clot quality qualitative tests such as VHAs are used to guide BCT/HAT [156]. In a prospective audit and retrospective extraction of data on major obstetric hemorrhage, ROTEM FIBTEM A5 was used to guide fibrinogen concentrate administration to 203 women experiencing PPH due to genital tract and surgical trauma including but not limited to uterine rupture and uterine inversion. When using the ROTEM-algorithm in this study instead of shock packs, there were significant decreases in rates of ICU admission, TACO, and morbidity. There was also a reduced usage of FFP from a median of 4 units to 0 units of FFP. The use of VHAs in these patients greatly improved clinical outcomes while guiding optimal use of blood products [76].

During a Cesarean (C-)section operation and for as long as 6–10 h post-operatively, fibrinogen and fibrin are degraded, while plasminogen activators and fibrin degradation products increase [157]. C-section rates have increased to as high as 25–30% in many areas of the world, but especially in developing countries, and as it is widely accepted that undergoing C-section increases the probability of PPH, it is becoming increasingly important to identify and implement effective prevention and treatment for PPH following C-section [158,159]. One algorithm for the use of TEG in PPH including in C-section has been established for when blood loss exceeds 1000 mL. This sequence involves determining blood loss, obtaining baseline traditional labs, and then using TEG to monitor and guide treatment until bleeding is controlled [88]. For these patients who had a C-section, early appreciation of hemostatic changes indicative of coagulopathy are helpful. To this end, TEG reference ranges have been established preoperatively and postoperatively in pregnant women undergoing C-section with spinal anesthesia compared to non-pregnant women. MA and alpha angle were found to be more elevated in pregnant women, while LY30 was found to be decreased in pregnant women compared to those who were not pregnant. Such parameters may be useful for physicians monitoring C-section patients at increased risk of PPH [49,90,160].

### 4.7. Other Amniotic Anomolies

Other etiologies of PPH such as adherent and retained placenta can be managed by VHA guided BCT/HAT with improvements in outcomes with reduction of the number of blood components administered [76,110].

Placental retention is a cause of major obstetric hemorrhage after delivery whose treatment has been guided by VHAs [15,54,161]. Retained placenta specifically can go onto cause persistent uterine atony discussed previously [99]. In a study of 45 women with PPH, 17 of which had placental retention, the use of TEG provided more rapid and imperative clinical information about the hematological changes occurring with the bleeding. The TEG tracings in this study showed faster initiating of clotting (causing consumption of platelet and clotting factors) denoted by a shorter R time, decreased fibrin clot strength as seen with a decreased MA and alpha angle, and depressed fibrinolysis demonstrated by a decreased LY30 in patients with PPH compared to those without. Indications for specific blood product therapy were also obtained at an earlier point in pathology progression compared to traditional blood testing making the use of VHAs advantageous over the more traditional laboratory tests [54].

### 4.8. Inherited and Prepartum Acquired Coagulopathies

VHA guided BCT/HAT is utilized in pregnant patients who experience PPH and who have inherited or prepartum acquired coagulopathies [110,162].

Parturients with inherited disorders of coagulation are at an increased risk of bleeding following childbirth. Examples of such coagulopathies include but are not limited to Von Willebrand disease, Hemophilia A, Hemophilia B, and Factor XI deficiency. Von Willebrand factor (vWF) works to promote clotting by adhering to injured tissues and causing platelet aggregation [163,164]. There are three types of Von Willebrand disease: type 1 is a deficiency in vWF, type 2 results from functional losses or gains of vWF and can be further complicated with a quantitative deficiency, and type 3 is when there is no vWF present at all. Normally in pregnancy, vWF levels increase, which can cause normal vWF levels in parturients who have type 1 disease, decreasing their risk of PPH. Pregnant patients with type 2 and 3 disease are still at risk for PPH, as type 2 parturients have a functional mutation in their vWF even if total concentration increases, and type 3 parturients lack an increase in vWF as they are unable to produce it regardless of childbearing status [165,166]. VHAs are not sensitive to vWD; however, specialized TEG testing called ristoce-tin-enhanced TEG^®^ with Platelet Mapping (TEG^®^ P/M) can be used to assess vWD [162,167,168,169,170,171,172,173]. Hemophilia A is a severe deficiency in factor VIII while hemophilia B is a severe factor IX deficiency. Females are much more commonly carriers of these diseases while their incidence in females is quite rare [174]. Carriers, however, can still exhibit decreased levels of factor concentrations, putting them at an increased risk for PPH [175]. Factor XI deficiency, also known as hemophilia C, is an inherited bleeding disorder that is most common among Ashkenazi Jews [176]. The role of factor XI is not studied; however, its role appears to be both procoagulant and antifibrinolytic in nature meaning this disorder also puts parturients at an increased risk of PPH [177]. All these inherited coagulopathies that women may have while pregnant, put women at an increased risk of PPH with any delivery type and having a rapid test that quantifies these more complicated clotting cascades is imperative. When bleeding does occur after delivery, whole-blood POC tests such as TEG and ROTEM have been used in these populations providing clot kinetics in real time that CCTs fail to provide. In addition, intrinsic thromboelastometry (INTEM), EXTEM, and FIBTEM have been successfully used to guide treatment in these patients with PPH. INTEM is a specialized ROTEM assay that focuses on the intrinsic pathway. These are additional types of ROTEM involving the addition of additives to isolate the function of the intrinsic clotting pathway, extrinsic clotting pathway, and fibrinogen respectively [13,95,110].

Acquired coagulopathies are also a cause for PPH and include but are not limited to platelet disorders and fibrinogen deficiencies [96,110]. Platelet disorders can result from hypertensive disorders, gestational thrombocytopenia, or idiopathic thrombocytopenic purpura. The two most important characteristics to consider in a pregnant patient with a platelet disorder are if the condition is dynamic or stable and if the platelet function in and of itself is normal or abnormal [178]. TEG-PM involves the addition of adenosine diphosphate or arachidonic acid as an additional reagent and can be used to assess platelet function [33,179]. The adenosine diphosphate and arachidonic acid isolate platelet function by causing platelet aggregation. However, given its novelty, it still needs to be validated [180,181]. Once parameters have been established for TEG-PM, this technology will be an extremely valuable resource in diagnosing and guiding treatment for patients who acquire platelet disorders along with their PPH. The association between a VHA-based fibrinogen concentration and the traditional Clauss fibrinogen measurement is still under investigation; however, a pooled analysis by Peng et al. showed a correlation plot where a Clause fibrinogen value of 1.5 g/L equated to a TEG-Functional Fibrinogen (TEG-FF) MA of 12 mm [182]. This plot did contain wide dispersion of experimental points and may have overestimated fibrinogen concentration for numerous reasons including platelet inhibitor use, high platelet count, and specific test used to run analysis [96]. While anticoagulant use is not exceedingly common in pregnancy, the use of low-molecular-weight heparin has begun to increase in use owing to the fact that fifty percent of deadly thrombotic events in patients are due to an inherited thrombophilia [183].

## 5. Discussion

The incidence of PPH is on the rise. However, the circumstances under which massive transfusion protocols are initiated are not well defined. Blood loss during childbirth is generally not well estimated, making it a difficult criterion to use. Beyond that, the recognition of “secondary PPH” poses a challenge as the timing is different from that of classic PPH. Finally, the CCTs used to anticipate potential need for massive transfusion are not dependable, nor are they suitable for a POC context. With the identification of the downfalls of the current standard of care CCTs, there has been an upsurge in literature regarding the use of VHAs for identification and treatment of PPH. Most of this new information has been derived from trauma literature, which has utilized VHAs for several decades. Table 1 provides a chronological description of significant studies excluding reviews covering the use of VHAs for PPH resuscitation.

The current strategy for managing patients with PPH combines the use of clinical prediction guidelines, CCTs, and VHAs. These protocols are used to define and treat PPH. The coagulopathy present during PPH cannot be satisfactorily categorized by CCTs alone. Low fibrinogen levels have been correlated with the development of PPH, but CCTs are not reliable or timely enough for sole use in identification or as guidance for treatment in those situations [53,71,72]. In other circumstances involving hemorrhage, such as trauma, the same has been found to be true, leading to adoption of the use of VHAs for better information and guidance of treatment. With the development of literature in the field of trauma, obstetrics has begun to utilize similar strategies by including VHAs to help recognize and treat PPH. Whereas there has been impressive advancement within the trauma literature, that which has been created by obstetricians has not shown as dramatic an expansion. While TIC will reliably present within seconds to hours following the inciting event, the different etiologies of PPH have varied presentations and timeframes. In cases of AFE, coagulopathy is more immediate and severe than in patients experiencing hemorrhage due to uterine atony; however, both patients require timely recognition of their pathology recognized by VHAs. There will be continued research into the application of TEG and ROTEM for PPH. This paper has served to outline the history of VHA research and its application in the field of obstetrics. We predict an increase in the evidence supporting the use of VHAs as their benefits to patient care and outcomes in obstetrics become more apparent, leading to implementation within the global clinical setting. Already there has been a significant evolution of using VHAs to reduce the incidence of massive postpartum hemorrhage as demonstrated by the most recent publication of the seminal experience of the national quality improvement project entitled: Reduction in massive postpartum haemorrhage and red blood cell transfusion during a national quality improvement project, Obstetric Bleeding Strategy for Wales, OBS Cymru: an observational study [17,184].

## Figures and Tables

**Table 1:** Review of salient literature regarding VHAs and their utility in PPH.

Reference	Study Design (VHA Used)	No. of Patients	Conclusions
Huissoud 2009 [52]	Prospective Observational (ROTEM)	54 Controls 37 PPH	(1) ROTEM parameters may be used to define transfusion thresholds. (2) FIBTEM provided early detection of decreases in fibrinogen.
Hill 2012 [88]	Prospective Observational (TEG)	57 Healthy Pre-Elective C-sections	(1) Non-activated assays produced different reference values than activated (rapid) assays for three TEG assays studied, thus; (2) TEG algorithms may be useful in PPH, but the reference TEG assay must be consistent or specified to the operator.
Collins 2014 [25]	Prospective Observational Study (ROTEM)	356 women with PPH	(1) Fibrinogen and FIBTEM POCTs are useful in predicting progression to severe bleeds. (2) Prolonged bleeding was exhibited with lower FIBTEM and fibrinogen levels. (3) Available within 10 min, FIBTEM A5 is an independent indicator of progression to severe bleeding.
Farber 2014 [85]	In vitro hemodilution model (TEG)	20 healthy parturients with uncomplicated pregnancies	(1) TEG variables are not significantly affected by differing hemodilutions.
Karlsson 2014 [54]	Prospective Observational (TEG)	45 women with PPH 49 women with blood loss <600 mL	(1) Both TEG and CCTs demonstrated impaired hemostasis during MOH after an estimated blood loss of 2000 mL. (2) CCTs provide more specific information on reason for impaired hemostasis, but TEG provides faster feedback to the medical team about hemostasis to guide intervention.
Wang 2014 [86]	Randomized Controlled Study (TEG)	190 women with pathological pregnancies 75 women with normal pregnancies	(1) Non-hemorrhaging patients exhibit a smaller MA than patients with preeclampsia. (2) Thrombosis patients showed a shorter R time and a larger MA than patients that were not hemorrhaging. (3) PPH risk can be identified accurately and quickly in patients with various pathological pregnancies.
Mallaiah 2015 [55]	Prospective Sequential phased Comparative study? ROTEM	42 patients with PPH	(1) The proposed algorithm allows for a reassessment of the response administration of fibrinogen replacement therapy, thus; (2) The algorithm allows for significant reduction in the total usage of blood products.
Wikkelso 2015 [57]	Randomized control trial	249 patients with PPH	(1) Fibrinogen concentrate is not an effective pre-emptive treatment for normofibrinoginaemic patients with PPH.
Shreeve 2016 [58]	Observational, cross-sectional/longitudinal study (TEG)	112 pregnant women	(1) Thromboelastography provided more clearly established reference ranges throughout pregnancy, labor, and the immediate postpartum period. (2) Thromboelastography can reliably identify changes in the mechanics of a clot. (3) TEG is advantageous to guiding BCT and is a useful tool in massive blood transfusion.
Barinov 2017 [81]	Open controlled trial (TEG)	119 women with PPH	(1) The combination of analysis of blood with TEG and compression of the uterine wall and uterine cavity draining via intrauterine balloon tamponade proves to be a valuable tool for fertility sparing treatment of obstetric hemorrhage.
Collins 2017 [59]	Double-blind randomized controlled trial (ROTEM)	55 women with PPH	(1) PPH outcomes are not improved by infusion of fibrinogen concentrate when FIBTEM produces an A5 < 15 mm. (2) FIBTEM A5 values over 12 mm or fibrinogen values of a concentration greater than 2 g/L are sufficient for hemostasis and infusion is not required.
Kaufner 2017 [82]	Prospective Observational pilot study (ROTEM)	217 healthy pregnant women	(1) Elevated prepartum fibrinogen levels are not associated with a reduced risk of PPH. (2) ROTEM is not a predictive instrument for postpartum blood loss.
Roberts 2018 [79]	A secondary analysis of the WOMAN trial (ROTEM)	167 women with PPH	(1) In a cohort of Nigerian women, ROTEM evidence of hyperfibrinolysis occurs in 23% of women with PPH.
Snegovskikh 2018 [3]	Retrospective cohort study (ROTEM)	86 patients with severe PPH	(1) VHA-based protocols were associated with improved patient outcomes, reduced blood product replacement, and reduced cost of care compared to more traditional empiric protocols.
Gillissen 2019 [75]	Prospective observational cohort study (ROTEM)	23 patients with PPH	(1) FIBTEM assays must always use device-specific reference, as CT values from both Sigma and Delta ROTEM lacked correlation. (2) Amplitudes measured in EXTEM, INTEM, and APTEM obtained from both Delta and Sigma ROTEM were similar.
Gootjes 2019 [21]	Prospective single-center study (ROTEM)	312 pregnant women	(1) Pregnant women of different ethnicities show no significant differences in their coagulation cascades. (2) Fibrinogen delivery based on FIBTEM parameters can efficiently stop bleeding, especially in PPH.
McNamara 2019 [76]	Observational study (ROTEM)	893 women with PPH 52 control patients	(1) ROTEM analysis demonstrates that coagulopathy cannot be predicted solely by blood loss because it is not observed in 100% of women who suffer from obstetric hemorrhage. (2) Placental abruption causes a more severe coagulopathy, necessitating a higher dosage of fibrinogen concentrate than in other cases of bleeding. (3) A ROTEM-guided fibrinogen concentrate algorithm resulted in a decrease in total blood products transfused, decreased transfusion-associated circulatory overload.
Toffaletti 2019 [77]	Retrospective analysis (ROTEM)	100 sets of EXTEM and FIBTEM data from patients undergoing operations for PPH	(1) EXTEM A10 correlates strongly to the EXTEM MCF, fibrinogen level, and platelet count. (2) This study supports the exclusive use of the A10 for POC diagnosis and therapeutic decisions in PPH situations. (3) The FIBTEM alpha angle and A10 is correlated with fibrinogen levels; these parameters could provide an earlier indication of the fibrinogen status of the patient and guide POC fibrinogen supplementation.
Arnolds 2020 [74]	Retrospective Single-Center Observational Study (TEG)	118 women with PPH	(1) Elevations in kaolin TEG LY30 may have been related to platelet-mediated clot retraction instead of fibrinolysis. (2) Fibrinolysis must be differentiated from clot retraction to detect elevated activity of fibrinolysis.
Rigouzzo 2020 [2]	Retrospective Cohort Analysis (TEG)	98 women with PPH	(1) During PPH, TEG parameters provided reliable detection of hypofibrinogenemia and thrombocytopenia, confirming the utility of TEG for rapid hemostasis assessment during PPH.

## References

[1] Say L., Chou D., Gemmill A., Tunçalp Ö., Moller A.B., Daniels J., Gülmezoglu A.M., Temmerman M., Alkema L. (2014). Global causes of maternal death: A WHO systematic analysis. Lancet Glob. Health.

[2] Rigouzzo A., Louvet N., Favier R., Ore M.-V., Piana F., Girault L., Farrugia M., Sabourdin N., Constant I. (2020). Assessment of coagulation by thromboelastography during ongoing postpartum hemorrhage: A retrospective cohort analysis. Anesth. Analg..

[3] Snegovskikh D., Souza D., Walton Z., Dai F., Rachler R., Garay A., Snegovskikh V.V., Braveman F.R., Norwitz E.R. (2018). Point-of-care viscoelastic testing improves the outcome of pregnancies complicated by severe postpartum hemorrhage. J. Clin. Anesth..

[4] Kramer M.S., Berg C., Abenhaim H., Dahhou M., Rouleau J., Mehrabadi A., Joseph K.S. (2013). Incidence, risk factors, and temporal trends in severe postpartum hemorrhage. Am. J. Obstet. Gynecol..

[5] Henriquez D.D.C.A., Bloemenkamp K.W.M., van der Bom J.G. (2018). Management of postpartum hemorrhage: How to improve maternal outcomes?. J. Thromb. Haemost..

[6] Dahlke J.D., Mendez-Figueroa H., Maggio L., Hauspurg A.K., Sperling J.D., Chauhan S.P., Rouse D.J. (2015). Prevention and management of postpartum hemorrhage: A comparison of 4 national guidelines. Am. J. Obstet. Gynecol..

[7] Guasch E., Gilsanz F. (2016). Massive obstetric hemorrhage: Current approach to management. Med. Intensiv..

[8] Munoz M., Stensballe J., Ducloy-Bouthors A.S., Bonnet M.P., De Robertis E., Fornet I., Goffinet F., Hofer S., Holzgreve W., Manrique S. (2019). Patient blood management in obstetrics: Prevention and treatment of postpartum haemorrhage. A NATA consensus statement. Blood Transfus..

[9] Leung Y., Sgroi J., Vaughan J., Pettigrew I., Jacobson T., Page I., Regan J., Skidmore C., Hui L., White B. (2017). Management of Postpartum Haemorrhage (PPH).

[10] Shields L.E., Goffman D., Caughey A.B. (2017). Practice Bulletin No. 183: Postpartum Hemorrhage. Obstet. Gynecol..

[11] Mavrides E., Allard S., Chandraharan E., Collins P., Green L., Hunt B.J., Riris S., Thomson A.J. (2016). Prevention and management of postpartum haemorrhage. BJOG.

[12] Gillissen A., van den Akker T., Caram-Deelder C., Henriquez D., Bloemenkamp K.W.M., de Maat M.P.M., van Roosmalen J.J.M., Zwart J.J., Eikenboom J., van der Bom J.G. (2018). Coagulation parameters during the course of severe postpartum hemorrhage: A nationwide retrospective cohort study. Blood Adv..

[13] de Lange N.M., Lancé M.D., de Groot R., Beckers E.A., Henskens Y.M., Scheepers H.C. (2012). Obstetric hemorrhage and coagulation: An update. Thromboelastography, thromboelastometry, and conventional coagulation tests in the diagnosis and prediction of postpartum hemorrhage. Obstet. Gynecol. Surv..

[14] Likis F.E., Sathe N.A., Morgans A.K., Hartmann K.E., Young J.L., Carlson-Bremer D., Schorn M., Surawicz T., Andrews J. (2015). Management of Postpartum Hemorrhage. Eff. Health Care Progr. Comp. Eff. Rev..

[15] Allard S., Green L., Hunt B.J. (2014). How we manage the haematological aspects of major obstetric haemorrhage. Br. J. Haematol..

[16] Collins P., Abdul-Kadir R., Thachil J. (2016). Management of coagulopathy associated with postpartum hemorrhage: Guidance from the SSC of the ISTH. J. Thromb. Haemost..

[17] Collins P.W., Bell S.F., de Lloyd L., Collis R.E. (2018). Management of postpartum haemorrhage: From research into practice, a narrative review of the literature and the Cardiff experience. Int. J. Obstet. Anesth..

[18] Hunt B.J., Lyons G. (2005). Thromboelastography should be available in every labour ward. Int. J. Obstet. Anesth..

[19] Butwick A., Lyell D., Goodnough L. (2020). How do I manage severe postpartum hemorrhage?. Transfusion.

[20] Gehrie E.A., Baine I., Booth G.S., Education Committee of the Academy of Clinical Laboratory Physicians and Scientists (2016). Pathology Consultation on Viscoelastic Studies of Coagulopathic Obstetrical Patients. Am. J. Clin. Pathol..

[21] Gootjes D.V., Kuipers I., Thomassen B.J., Verheul R.J., de Vries S., Mingelen W., van Dunné F.M., Ponjee G.A. (2019). ROTEM reference ranges in a pregnant population from different nationalities/ethnic backgrounds. Int. J. Lab. Hematol..

[22] van der Bom J.G. (2014). Rotem in postpartum hemorrhage. Blood.

[23] Hartmann J., Walsh M., Grisoli A., Thomas A.V., Shariff F., McCauley R., Lune S.V., Zackariya N., Patel S., Farrell M.S. (2020). Diagnosis and Treatment of Trauma-Induced Coagulopathy by Viscoelastography. Semin. Thromb. Hemost..

[24] Schochl H., Voelckel W., Grassetto A., Schlimp C.J. (2013). Practical application of point-of-care coagulation testing to guide treatment decisions in trauma. J. Trauma Acute Care Surg..

[25] Collins P.W., Lilley G., Bruynseels D., Laurent D.B., Cannings-John R., Precious E., Hamlyn V., Sanders J., Alikhan R., Rayment R. (2014). Fibrin-based clot formation as an early and rapid biomarker for progression of postpartum hemorrhage: A prospective study. Blood.

[26] Orlikowski C.E., Rocke D.A., Murray W.B., Gouws E., Moodley J., Kenoyer D.G., Byrne S. (1996). Thrombelastography changes in pre-eclampsia and eclampsia. Br. J. Anaesth..

[27] Oudghiri M., Keita H., Kouamou E., Boutonnet M., Orsini M., Desconclois C., Mandelbrot L., Daures J.-P., Stépanian A., Peynaud-Debayle E. (2011). Reference values for rotation thromboelastometry (ROTEM®) parameters following non-haemorrhagic deliveries. Correlations with standard haemostasis parameters. Thromb. Haemost..

[28] Collis R.E., Collins P.W. (2015). Haemostatic management of obstetric haemorrhage. Anaesthesia.

[29] Hurwich M., Zimmer D., Guerra E., Evans E., Shire T., Abernathy M., Shreve J.T., Kolettis G.R., McCurdy M.T., Castellino F.J. (2016). A Case of Successful Thromboelastographic Guided Resuscitation after Postpartum Hemorrhage and Cardiac Arrest. J. Extra-Corpor. Technol..

[30] Monte S., Lyons G. (2002). Peripartum management of a patient with Glanzmann’s thrombasthenia using Thrombelastograph®. Br. J. Anaesth..

[31] Othman M., Falcon B.J., Kadir R. (2010). Global hemostasis in pregnancy: Are we using thromboelastography to its full potential?. Semin. Thromb. Hemost..

[32] Stocks G. (2011). Monitoring transfusion requirements in major obstetric haemorrhage: Out with the old and in with the new?. Int. J. Obstet. Anesth..

[33] Amgalan A., Allen T., Othman M., Ahmadzia H.K. (2020). Systematic review of viscoelastic testing (TEG/ROTEM) in obstetrics and recommendations from the Women's SSC of the ISTH. J. Thromb. Haemost..

[34] Hans G.A., Besser M.W. (2016). The place of viscoelastic testing in clinical practice. Br. J. Haematol..

[35] Kang Y.G., Martin D.J., Marquez J., Lewis J.H., Bontempo F.A., Shaw B.W., Starzl T.E., Winter P.M. (1985). Intraoperative changes in blood coagulation and thrombelastographic monitoring in liver transplantation. Anesth. Analg..

[36] Kaufmann C.R., Dwyer K.M., Crews J.D., Dols S.J., Trask A.L. (1997). Usefulness of thrombelastography in assessment of trauma patient coagulation. J. Trauma.

[37] Shore-Lesserson L., Manspeizer H.E., DePerio M., Francis S., Vela-Cantos F., Ergin M.A. (1999). Thromboelastography-guided transfusion algorithm reduces transfusions in complex cardiac surgery. Anesth. Analg..

[38] Rugeri L., Levrat A., David J.S., Delecroix E., Floccard B., Gros A., Allaouchiche B., Negrier C. (2007). Diagnosis of early coagulation abnormalities in trauma patients by rotation thrombelastography. J. Thromb. Haemost..

[39] Plotkin A.J., Wade C.E., Jenkins D.H., Smith K.A., Noe J.C., Park M.S., Perkins J.G., Holcomb J.B. (2008). A reduction in clot formation rate and strength assessed by thrombelastography is indicative of transfusion requirements in patients with penetrating injuries. J. Trauma.

[40] Schochl H., Frietsch T., Pavelka M., Jambor C. (2009). Hyperfibrinolysis after major trauma: Differential diagnosis of lysis patterns and prognostic value of thrombelastometry. J. Trauma.

[41] Johansson P.I. (2012). Coagulation monitoring of the bleeding traumatized patient. Curr. Opin. Anaesthesiol..

[42] Holcomb J.B., Minei K.M., Scerbo M.L., Radwan Z.A., Wade C.E., Kozar R.A., Gill B.S., Albarado R., McNutt M.K., Khan S. (2012). Admission rapid thrombelastography can replace conventional coagulation tests in the emergency department: Experience with 1974 consecutive trauma patients. Ann. Surg..

[43] Moore H.B., Moore E.E., Liras I.N., Gonzalez E., Harvin J.A., Holcomb J.B., Sauaia A., Cotton B.A. (2016). Acute fibrinolysis shutdown after injury occurs frequently and increases mortality: A multicenter evaluation of 2540 severely injured patients. JACS.

[44] Holcomb J.B., Tilley B.C., Baraniuk S., Fox E.E., Wade C.E., Podbielski J.M., del Junco D.J., Brasel K.J., Bulger E.M., Callcut R.A. (2015). Transfusion of plasma, platelets, and red blood cells in a 1:1:1 vs a 1:1:2 ratio and mortality in patients with severe trauma: The PROPPR randomized clinical trial. JAMA.

[45] Sperry J.L., Guyette F.X., Brown J.B., Yazer M.H., Triulzi D.J., Early-Young B.J., Adams P.W., Daley B.J., Miller R.S., Harbrecht B.G. (2018). Prehospital plasma during air medical transport in trauma patients at risk for hemorrhagic shock. N. Engl. J. Med..

[46] Moore H.B., Moore E.E., Chapman M.P., McVaney K., Bryskiewicz G., Blechar R., Chin T., Burlew C.C., Pieracci F., West F.B. (2018). Plasma-first resuscitation to treat haemorrhagic shock during emergency ground transportation in an urban area: A randomised trial. Lancet.

[47] Guyette F.X., Brown J.B., Zenati M.S., Early-Young B.J., Adams P.W., Eastridge B.J., Nirula R., Vercruysse G.A., O’Keeffe T., Joseph B. (2021). Tranexamic acid during prehospital transport in patients at risk for hemorrhage after injury: A double-blind, placebo-controlled, randomized clinical trial. JAMA Surg..

[48] Lee J., Eley V., Wyssusek K., Kimble R., Way M., Coonan E., Cohen J., Rowell J., van Zundert A. (2020). Baseline parameters for rotational thromboelastometry in healthy labouring women: A prospective observational study. BJOG.

[49] Lee J., Eley V.A., Wyssusek K.H., Coonan E., Way M., Cohen J., Rowell J., van Zundert A.A. (2019). Baseline parameters for rotational thromboelastometry (ROTEM®) in healthy women undergoing elective caesarean delivery: A prospective observational study in Australia. Int. J. Obstet. Anesth..

[50] Lee J., Wyssusek K.H., Kimble R.M.N., Way M., van Zundert A.A., Cohen J., Rowell J., Eley V.A. (2020). Baseline parameters for rotational thromboelastometry (ROTEM®) in healthy pregnant Australian women: A comparison of labouring and non-labouring women at term. Int. J. Obstet. Anesth..

[51] Moopanar S., Naidu J., Moodley E., Gouws D. (1997). Thromboelastography in abruptio placentae. J. Obstet. Gynaecol..

[52] Huissoud C., Carrabin N., Audibert F., Levrat A., Massignon D., Berland M., Rudigoz R.C. (2009). Bedside assessment of fibrinogen level in postpartum haemorrhage by thrombelastometry. BJOG.

[53] Armstrong S., Fernando R., Ashpole K., Simons R., Columb M. (2011). Assessment of coagulation in the obstetric population using ROTEM® thromboelastometry. Int. J. Obstet. Anesth..

[54] Karlsson O., Jeppsson A., Hellgren M. (2014). Major obstetric haemorrhage: Monitoring with thromboelastography, laboratory analyses or both?. Int. J. Obstet. Anesth..

[55] Mallaiah S., Barclay P., Harrod I., Chevannes C., Bhalla A. (2015). Introduction of an algorithm for ROTEM-guided fibrinogen concentrate administration in major obstetric haemorrhage. Anaesthesia.

[56] McNamara H., Mallaiah S., Barclay P., Chevannes C., Bhalla A. (2015). Coagulopathy and placental abruption: Changing management with ROTEM-guided fibrinogen concentrate therapy. Int. J. Obstet. Anesth..

[57] Wikkelso A.J. (2015). The role of fibrinogen and haemostatic assessment in postpartum haemorrhage: Preparations for a randomised controlled trial. Dan. Med. J..

[58] Shreeve N.E., Barry J.A., Deutsch L.R., Gomez K., Kadir R.A. (2016). Changes in thromboelastography parameters in pregnancy, labor, and the immediate postpartum period. Int. J. Gynaecol. Obstet..

[59] Collins P.W., Cannings-John R., Bruynseels D., Mallaiah S., Dick J., Elton C., Weeks A., Sanders J., Aawar N., Townson J. (2017). Viscoelastometric-guided early fibrinogen concentrate replacement during postpartum haemorrhage: OBS2, a double-blind randomized controlled trial. Br. J. Anaesth..

[60] Collins P.W., Cannings-John R., Bruynseels D., Mallaiah S., Dick J., Elton C., Weeks A., Sanders J., Aawar N., Townson J. (2017). Viscoelastometry guided fresh frozen plasma infusion for postpartum haemorrhage: OBS2, an observational study. Br. J. Anaesth..

[61] Walsh M., Thomas S., Kwaan H., Aversa J., Anderson S., Sundararajan R., Zimmer D., Bunch C., Stillson J., Draxler D. (2020). Modern methods for monitoring hemorrhagic resuscitation in the United States: Why the delay?. J. Trauma Acute Care Surg..

[62] Bareille M., Hardy M., Douxfils J., Roullet S., Lasne D., Levy J.H., Stépanian A., Susen S., Frère C., Lecompte T. (2021). Viscoelastometric Testing to Assess Hemostasis of COVID-19: A Systematic Review. J. Clin. Med..

[63] Walsh M.M., Khan R., Kwaan H.C., Neal M.D. (2021). Fibrinolysis Shutdown in COVID-19-Associated Coagulopathy: A Crosstalk among Immunity, Coagulation, and Specialists in Medicine and Surgery. J. Am. Coll Surg..

[64] Stillson J.E., Bunch C.M., Gillespie L., Khan R., Wierman M., Pulvirenti J., Phyu H., Anderson S., Al-Fadhl M., Thomas A.V. (2021). Thromboelastography-Guided Management of Anticoagulated COVID-19 Patients to Prevent Hemorrhage. Semin. Thromb. Hemost..

[65] Bugaev N., Como J.J., Golani G., Freeman J.J., Sawhney J.S., Vatsaas C.J., Yorkgitis B.K., Kreiner L.A., Garcia N.M., Aziz H.A. (2020). Thromboelastography and Rotational Thromboelastometry in Bleeding Patients with Coagulopathy: Practice Management Guideline from the Eastern Association for the Surgery of Trauma. J. Trauma Acute Care Surg..

[66] Bunch C.M., Thomas A.V., Stillson J.E., Gillespie L., Khan R.Z., Zackariya N., Shariff F., Al-Fadhl M., Mjaess N., Miller P.D. (2021). Preventing Thrombohemorrhagic Complications of Heparinized COVID-19 Patients Using Adjunctive Thromboelastography: A Retrospective Study. J. Clin. Med..

[67] Abdul-Kadir R., McLintock C., Ducloy A.S., El-Refaey H., England A., Federici A.B., Grotegut C.A., Halimeh S., Herman J.H., Hofer S. (2014). Evaluation and management of postpartum hemorrhage: Consensus from an international expert panel. Transfusion.

[68] Bonnet M.P., Benhamou D. (2016). Management of postpartum haemorrhage. F1000Research.

[69] Gülmezoglu A.M., Souza J.P., Mathai M., Abalos E., Díaz V., Hezelgrave N., Watananirun K. (2012). WHO Recommendations for the Prevention and Treatment of Postpartum Haemorrhage.

[70] Pavord S., Maybury H. (2015). How I treat postpartum hemorrhage. Blood.

[71] Charbit B., Mandelbrot L., Samain G., Baron G., Haddaoui B., Keita H., Sibony O., Mahieu-Caputo D., Hurtaud-Roux M.F., Huisse M.G. (2007). The decrease of fibrinogen is an early predictor of the severity of postpartum hemorrhage. J. Thromb. Haemost..

[72] de Lloyd L., Bovington R., Kaye A., Collis R.E., Rayment R., Sanders J., Rees A., Collins P.W. (2011). Standard haemostatic tests following major obstetric haemorrhage. Int. J. Obstet. Anesth..

[73] Curry N.S., Davenport R., Pavord S., Mallett S.V., Kitchen D., Klein A.A., Maybury H., Collins P.W., Laffan M. (2018). The use of viscoelastic haemostatic assays in the management of major bleeding: A British Society for Haematology Guideline. Br. J. Haematol..

[74] Arnolds D.E., Scavone B.M. (2020). Thromboelastographic assessment of fibrinolytic activity in postpartum hemorrhage: A retrospective single-center observational study. Anesth. Analg..

[75] Gillissen A., van den Akker T., Caram-Deelder C., Henriquez D.D., Bloemenkamp K.W., Eikenboom J., van der Bom J.G., de Maat M.P. (2019). Comparison of thromboelastometry by ROTEM® Delta and ROTEM® Sigma in women with postpartum haemorrhage. Scand. J. Clin. Lab. Invest..

[76] McNamara H., Kenyon C., Smith R., Mallaiah S., Barclay P. (2019). Four years’ experience of a ROTEM((R))-guided algorithm for treatment of coagulopathy in obstetric haemorrhage. Anaesthesia.

[77] Toffaletti J.G., Buckner K.A. (2019). Use of Earlier-Reported Rotational Thromboelastometry Parameters to Evaluate Clotting Status, Fibrinogen, and Platelet Activities in Postpartum Hemorrhage Compared to Surgery and Intensive Care Patients. Anesth. Analg..

[78] Wang M., Hu Z., Cheng Q.X., Xu J., Liang C. (2019). The ability of thromboelastography parameters to predict severe pre-eclampsia when measured during early pregnancy. Int. J. Gynaecol. Obstet..

[79] Roberts I., Shakur H., Fawole B., Kuti M., Olayemi O., Bello A., Ogunbode O., Kotila T., Aimakhu C.O., Olutogun T. (2018). Haematological and fibrinolytic status of Nigerian women with post-partum haemorrhage. BMC Pregnancy Childbirth.

[80] Ahmadzia H.K., Lockhart E.L., Thomas S.M., Welsby I.J., Hoffman M.R., James A.H., Murtha A.P., Swamy G.K., Grotegut C.A. (2017). Using antifibrinolytics in the peripartum period–concern for a hypercoagulable effect?. J. Neonatal Perinatal. Med..

[81] Barinov S.V., Zhukovsky Y.G., Dolgikh V.T., Medyannikova I.V. (2017). Novel combined strategy of obstetric haemorrhage management during caesarean section using intrauterine balloon tamponade. J. Matern. Fetal Neonatal Med..

[82] Kaufner L., Henkelmann A., von Heymann C., Feldheiser A., Mickley L., Niepraschk-von Dollen K., Grittner U., Henrich W., Bamberg C. (2017). Can prepartum thromboelastometry-derived parameters and fibrinogen levels really predict postpartum hemorrhage?. J. Perinat. Med..

[83] Zhou J., Xin Y., Ding Q., Jiang L., Chen Y., Dai J., Lu Y., Wu X., Liang Q., Wang H. (2016). Thromboelastography predicts risks of obstetric complication occurrence in (hypo)dysfibrinogenemia patients under non-pregnant state. Clin. Exp. Pharmacol. Physiol..

[84] de Lange N.M., van Rheenen-Flach L.E., Lancé M.D., Mooyman L., Woiski M., Van Pampus E.C., Porath M., Bolte A.C., Smits L., Henskens Y.M. (2014). Peri-partum reference ranges for ROTEM® thromboelastometry. Br. J. Anaesth..

[85] Farber M.K., Sadana N., Kaufman R.M., Liu X., Kodali B.S. (2014). Transfusion ratios for postpartum hemodilutional coagulopathy: An in vitro thromboelastographic model. Am. J. Obstet. Gynecol..

[86] Wang W., Wang A., Huang X., Jiang W., Jia X. (2014). Thromboelastography in women with pathological pregnancies: A preliminary study. Chin. Med. Sci. J..

[87] van Rheenen-Flach L.E., Zweegman S., Boersma F., Lenglet J.E., Twisk J.W., Bolte A.C. (2013). A prospective longitudinal study on rotation thromboelastometry in women with uncomplicated pregnancies and postpartum. Aust. N. Z. J. Obstet. Gynaecol..

[88] Hill J.S., Devenie G., Powell M. (2012). Point-of-care testing of coagulation and fibrinolytic status during postpartum haemorrhage: Developing a thrombelastography(R)-guided transfusion algorithm. Anaesth. Intensive Care.

[89] Karlsson O., Sporrong T., Hillarp A., Jeppsson A., Hellgren M. (2012). Prospective longitudinal study of thromboelastography and standard hemostatic laboratory tests in healthy women during normal pregnancy. Anesth. Analg..

[90] Macafee B., Campbell J.P., Ashpole K., Cox M., Matthey F., Acton L., Yentis S.M. (2012). Reference ranges for thromboelastography (TEG((R)) ) and traditional coagulation tests in term parturients undergoing caesarean section under spinal anaesthesia*. Anaesthesia.

[91] Huissoud C., Carrabin N., Benchaib M., Fontaine O., Levrat A., Massignon D., Touzet S., Rudigoz R.C., Berland M. (2009). Coagulation assessment by rotation thrombelastometry in normal pregnancy. Thromb. Haemost..

[92] Smith R., Campbell-Owen T., Maybury H., Pavord S., Waugh J. (2009). Thromboelastography and peripartum coagulation profiles associated with caesarean section delivery. Obstet. Med..

[93] Karlsson O. (2017). Experience of point-of-care devices in obstetrical care. Semin. Thromb. Hemost..

[94] Sharma S.K., Vera R.L., Stegall W.C., Whitten C.W. (1997). Management of a postpartum coagulopathy using thrombelastography. J. Clin. Anesth..

[95] Carroll R.C., Craft R.M., Chavez J.J., Snider C.C., Kirby R.K., Cohen E. (2008). Measurement of functional fibrinogen levels using the Thrombelastograph. J. Clin. Anesth..

[96] Ranucci M., Di Dedda U., Baryshnikova E. (2020). Trials and tribulations of viscoelastic-based determination of fibrinogen concentration. Anesth. Analg..

[97] Westbrook A.J., Olsen J., Bailey M., Bates J., Scully M., Salamonsen R.F. (2009). Protocol based on thromboelastograph (TEG) out-performs physician preference using laboratory coagulation tests to guide blood replacement during and after cardiac surgery: A pilot study. Heart Lung Circ..

[98] McDonnell N.J., Browning R. (2018). How to replace fibrinogen in postpartum haemorrhage situations? (Hint: Don’t use FFP!). Int. J. Obstet. Anesth..

[99] Bell S.F., Rayment R., Collins P.W., Collis R. (2010). The use of fibrinogen concentrate to correct hypofibrinogenaemia rapidly during obstetric haemorrhage. Int. J. Obstet. Anesth..

[100] Glover N., Collis R., Collins P. (2010). Fibrinogen concentrate use during major obstetric haemorrhage. Anaesthesia.

[101] Wikkelsø A.J., Edwards H.M., Afshari A., Stensballe J., Langhoff-Roos J., Albrechtsen C., Ekelund K., Hanke G., Secher E.L., Sharif H.F. (2015). Pre-emptive treatment with fibrinogen concentrate for postpartum haemorrhage: Randomized controlled trial. Br. J. Anaesth..

[102] Solomon C., Collis R.E., Collins P.W. (2012). Haemostatic monitoring during postpartum haemorrhage and implications for management. Br. J. Anaesth..

[103] Alport E.C., Callum J.L., Nahirniak S., Eurich B., Hume H.A. (2008). Cryoprecipitate use in 25 Canadian hospitals: Commonly used outside of the published guidelines. Transfusion.

[104] Walsh M., Moore E.E., Moore H.B., Thomas S., Kwaan H.C., Speybroeck J., Marsee M., Bunch C.M., Stillson J., Thomas A.V. (2021). Whole Blood, Fixed Ratio, or Goal-Directed Blood Component Therapy for the Initial Resuscitation of Severely Hemorrhaging Trauma Patients: A Narrative Review. J. Clin. Med..

[105] Mallaiah S., Chevannes C., McNamara H., Barclay P. (2015). A reply. Anaesthesia.

[106] McNamara H., Mallaiah S. (2019). Managing coagulopathy following PPH. Best Pract. Res. Clin. Obstet. Gynaecol..

[107] Haas T., Gorlinger K., Grassetto A., Agostini V., Simioni P., Nardi G., Ranucci M. (2014). Thromboelastometry for guiding bleeding management of the critically ill patient: A systematic review of the literature. Minerv. Anestesiol..

[108] Agarwal S., Laycock H.C. (2020). The debate ROTEMs on–the utility of point-of-care testing and fibrinogen concentrate in postpartum haemorrhage. Anaesthesia.

[109] Ekelund K., Hanke G., Stensballe J., Wikkelsoe A., Albrechtsen C.K., Afshari A. (2015). Hemostatic resuscitation in postpartum hemorrhage—A supplement to surgery. Acta Obstet. Gynecol. Scand..

[110] Katz D., Beilin Y. (2015). Disorders of coagulation in pregnancy. Br. J. Anaesth..

[111] Walsh M., Shreve J., Thomas S., Moore E., Moore H., Hake D., Pohlman T., Davis P., Ploplis V., Piscoya A. (2017). Fibrinolysis in trauma: “myth,” “reality,” or “something in between”. Semin. Thromb. Hemost..

[112] Kozek-Langenecker S.A., Afshari A., Albaladejo P., Santullano C.A.A., De Robertis E., Filipescu D.C., Fries D., Goerlinger K., Haas T., Imberger G. (2013). Management of severe perioperative bleeding: Guidelines from the European Society of Anaesthesiology. Eur. J. Anaesthesiol..

[113] Moore E.E., Moore H.B., Chapman M.P., Gonzalez E., Sauaia A. (2018). Goal-directed hemostatic resuscitation for trauma induced coagulopathy: Maintaining homeostasis. J. Trauma Acute Care Surg..

[114] Dragan B., Adamik B., Burzynska M., Dragan S.L., Gozdzik W. (2021). Platelet Receptor Activity for Predicting Survival in Patients with Intracranial Bleeding. J. Clin. Med..

[115] Can M.M., Tanboğa I.H., Türkyilmaz E., Karabay C.Y., Akgun T., Koca F., Tokgoz H.C., Keles N., Ozkan A., Bezgin T. (2010). The risk of false results in the assessment of platelet function in the absence of antiplatelet medication: Comparison of the PFA-100, multiplate electrical impedance aggregometry and verify now assays. Thromb. Res..

[116] Riojas C.M., Ekaney M.L., Ross S.W., Cunningham K.W., Furay E.J., Brown C.V., Evans S.L. (2021). Platelet Dysfunction after Traumatic Brain Injury: A Review. J. Neurotrauma.

[117] Olldashi F., Kerçi M., Zhurda T., Ruçi K., Banushi A., Traverso M.S., Jiménez J., Balbi J., Dellera C., Svampa S. (2010). Effects of tranexamic acid on death, vascular occlusive events, and blood transfusion in trauma patients with significant haemorrhage (CRASH-2): A randomised, placebo-controlled trial. Lancet.

[118] Shakur H., Roberts I., Fawole B., Chaudhri R., El-Sheikh M., Akintan A., Qureshi Z., Kidanto H., Vwalika B., Abdulkadir A. (2017). Effect of early tranexamic acid administration on mortality, hysterectomy, and other morbidities in women with post-partum haemorrhage (WOMAN): An international, randomised, double-blind, placebo-controlled trial. Lancet.

[119] Roberts I., Shakur H., Coats T., Hunt B., Balogun E., Barnetson L., Cook L., Kawahara T., Perel P., Prieto-Merino D. (2013). The CRASH-2 trial: A randomised controlled trial and economic evaluation of the effects of tranexamic acid on death, vascular occlusive events and transfusion requirement in bleeding trauma patients. Health Technol. Assess..

[120] Binz S., McCollester J., Thomas S., Miller J., Pohlman T., Waxman D., Shariff F., Tracy R., Walsh M. (2015). CRASH-2 Study of Tranexamic Acid to Treat Bleeding in Trauma Patients: A Controversy Fueled by Science and Social Media. J. Blood Transfus..

[121] Lockhart E. (2015). Postpartum hemorrhage: A continuing challenge. Hematol. Am. Soc. Hematol. Educ. Program..

[122] Moore E.E., Moore H.B., Gonzalez E., Sauaia A., Banerjee A., Silliman C.C. (2016). Rationale for the selective administration of tranexamic acid to inhibit fibrinolysis in the severely injured patient. Transfusion.

[123] Roberts I. (2016). Fibrinolytic shutdown: Fascinating theory but randomized controlled trial data are needed. Transfusion.

[124] Letson H.L., Dobson G.P. (2017). Tranexamic acid for post-partum haemorrhage in the WOMAN trial. Lancet.

[125] Walsh M., Thomas S., Moore E., Moore H., Piscoya A., Hake D., Son M., Pohlman T., Wegner J., Bryant J. (2017). Tranexamic Acid for Trauma Resuscitation in the United States of America. Semin. Thromb. Hemost..

[126] Jackson D.L., DeLoughery T.G. (2018). Postpartum hemorrhage: Management of massive transfusion. Obstet. Gynecol. Surv..

[127] Sentilhes L., Winer N., Azria E., Sénat M.-V., Le Ray C., Vardon D., Perrotin F., Desbrière R., Fuchs F., Kayem G. (2018). Tranexamic Acid for the Prevention of Blood Loss after Vaginal Delivery. N. Engl. J. Med..

[128] Shakur-Still H., Roberts I. (2018). Finding Better Ways to Prevent Postpartum Hemorrhage. N. Engl. J. Med..

[129] Myers S.P., Kutcher M.E., Rosengart M.R., Sperry J.L., Peitzman A.B., Brown J.B., Neal M.D. (2019). Tranexamic acid administration is associated with an increased risk of posttraumatic venous thromboembolism. J. Trauma Acute Care Surg..

[130] Tignanelli C.J., Napolitano L.M. (2019). The Fragility Index in Randomized Clinical Trials as a Means of Optimizing Patient Care. JAMA Surg..

[131] Walsh M., Grisoli A., Zackariya N., Thomas A.V., Sualeh A. (2020). Randomized controlled trials and Cochrane analyses versus precision—Based medicine for tranexamic acid and viscoelastic testing in trauma. ANZ J. Surg..

[132] Butwick A. (2020). Postpartum Hemorrhage: Wherefore Art Thou, Hyperfibrinolysis?. Anesth. Analg..

[133] Lier H., von Heymann C., Korte W., Schlembach D. (2018). Peripartum haemorrhage: Haemostatic aspects of the new German PPH guideline. Transfus. Med. Hemother..

[134] Breathnach F., Geary M. (2009). Uterine atony: Definition, prevention, nonsurgical management, and uterine tamponade. Semin. Perinatol..

[135] Lennox C., Marr L. (2014). Scottish Confidential Audit of Severe Maternal Morbidity: Reducing Avoidable Harm: 10th Annual Report.

[136] Chen Y., Huang P., Han C., Li J., Liu L., Zhao Z., Gao Y., Qin Y., Xu Q., Yan Y. (2021). Association of placenta-derived extracellular vesicles with pre-eclampsia and associated hypercoagulability: A clinical observational study. BJOG.

[137] Mello G., Parretti E., Marozio L., Pizzi C., Lojacono A., Frusca T., Facchinetti F., Benedetto C. (2005). Thrombophilia is significantly associated with severe preeclampsia: Results of a large-scale, case-controlled study. Hypertension.

[138] Mayrink J., Costa M.L., Cecatti J.G. (2018). Preeclampsia in 2018: Revisiting Concepts, Physiopathology, and Prediction. Sci. World J..

[139] Chaiworapongsa T., Yoshimatsu J., Espinoza J., Kim Y.M., Berman S., Edwin S., Yoon B.H., Romero R. (2002). Evidence of in vivo generation of thrombin in patients with small-for-gestational-age fetuses and pre-eclampsia. J. Matern. Fetal Neonatal Med..

[140] Eskild A., Vatten L.J. (2009). Abnormal bleeding associated with preeclampsia: A population study of 315,085 pregnancies. Acta Obstet. Gynecol. Scand..

[141] Lidan H., Jianbo W., Liqin G., Jifen H., Lin L., Xiuyan W. (2019). The Diagnostic Efficacy of Thrombelastography (TEG) in Patients with Preeclampsia and its Association with Blood Coagulation. Open Life Sci..

[142] Tanjung M.T., Siddik H.D., Hariman H., Koh S.C. (2005). Coagulation and fibrinolysis in preeclampsia and neonates. Clin. Appl. Thromb. Hemost..

[143] Balcı Ekmekçi Ö., Ekmekçi H., Güngör Z., Tüten A., Toprak M.S., Korkmaz M., Öncül M., Çalışkan O., Kucur M., Donma O. (2015). Evaluation of Lp-PLA2 mass, vitronectin and PAI-1 activity levels in patients with preeclampsia. Arch. Gynecol. Obstet..

[144] Naoum E.E., Leffert L.R., Chitilian H.V., Gray K.J., Bateman B.T. (2019). Acute fatty liver of pregnancy: Pathophysiology, anesthetic implications, and obstetrical management. Anesthesiology.

[145] Ch’Ng C.L., Morgan M., Hainsworth I., Kingham J.G.C. (2002). Prospective study of liver dysfunction in pregnancy in Southwest Wales. Gut.

[146] Mjahed K., Charra B., Hamoudi D., Noun M., Barrou L. (2006). Acute fatty liver of pregnancy. Arch. Gynecol. Obstet..

[147] Crochemore T., de Toledo Piza F.M., Silva E., Corrêa T.D. (2015). Thromboelastometry-guided hemostatic therapy: An efficacious approach to manage bleeding risk in acute fatty liver of pregnancy: A case report. J. Med. Case Rep..

[148] Kaur K., Bhardwaj M., Kumar P., Singhal S., Singh T., Hooda S. (2016). Amniotic fluid embolism. J. Anaesthesiol. Clin. Pharmacol..

[149] Tsunemi T., Oi H., Sado T., Naruse K., Noguchi T., Kobayashi H. (2012). An overview of amniotic fluid embolism: Past, present and future directions. Open Womens Health J..

[150] Levy J.H., Koster A., Quinones Q.J., Milling T.J., Key N.S. (2018). Antifibrinolytic therapy and perioperative considerations. Anesthesiology.

[151] Fudaba M., Tachibana D., Misugi T., Nakano A., Koyama M. (2021). Excessive fibrinolysis detected with thromboelastography in a case of amniotic fluid embolism: Fibrinolysis may precede coagulopathy. J. Thromb. Thrombolysis.

[152] Loughran J.A., Kitchen T.L., Sindhakar S., Ashraf M., Awad M., Kealaher E.J. (2019). Rotational thromboelastometry (ROTEM®)-guided diagnosis and management of amniotic fluid embolism. Int. J. Obstet. Anesth..

[153] Harnett M.J., Hepner D.L., Datta S., Kodali B.S. (2005). Effect of amniotic fluid on coagulation and platelet function in pregnancy: An evaluation using thromboelastography. Anaesthesia.

[154] Levin V.A., Villenueve J., Jiang X. (2018). Disseminated Intravascular Coagulation due to Amniotic Fluid Embolism in an Early Molar Pregnancy. Clin. Med. Rev. Case Rep..

[155] Rattray D.D., O’Connell C.M., Baskett T.F. (2012). Acute Disseminated Intravascular Coagulation in Obstetrics: A Tertiary Centre Population Review (1980 to 2009). J. Obstet. Gynaecol. Can..

[156] Neligan P.J., Laffey J.G. (2011). Clinical review: Special populations-critical illness and pregnancy. Crit. Care.

[157] Gai M.Y., Wu L.F., Su Q.F., Tatsumoto K. (2004). Clinical observation of blood loss reduced by tranexamic acid during and after caesarian section: A multi-center, randomized trial. Eur. J. Obstet. Gynecol. Reprod. Biol..

[158] Kambo I., Bedi N., Dhillon B.S., Saxena N.C. (2002). A critical appraisal of cesarean section rates at teaching hospitals in India. Int. J. Gynaecol. Obstet..

[159] Al-Zirqi I., Vangen S., Forsén L., Stray-Pedersen B. (2009). Effects of onset of labor and mode of delivery on severe postpartum hemorrhage. Am. J. Obstet. Gynecol..

[160] Sharma S.K., Philip J. (1997). The effect of anesthetic techniques on blood coagulability in parturients as measured by thromboelastography. Anesth. Analg..

[161] Balki M., Dhumne S., Kasodekar S., Carvalho J.C., Seaward G. (2008). Blood transfusion for primary postpartum hemorrhage: A tertiary care hospital review. J. Obstet. Gynaecol. Can..

[162] Ramiz S., Hartmann J., Young G., Escobar M.A., Chitlur M. (2019). Clinical utility of viscoelastic testing (TEG and ROTEM analyzers) in the management of old and new therapies for hemophilia. Am. J. Hematol..

[163] Reininger A.J., Heijnen H.F., Schumann H., Specht H.M., Schramm W., Ruggeri Z.M. (2006). Mechanism of platelet adhesion to von Willebrand factor and microparticle formation under high shear stress. Blood.

[164] Amorde R.W., Patel S.N., Pagel P.S. (2011). Management of labor and delivery of a patient with von Willebrand disease type 2a. Int. Anesthesiol. Clin..

[165] Silver R.M., Major H. (2010). Maternal coagulation disorders and postpartum hemorrhage. Clin. Obstet. Gynecol..

[166] Pacheco L.D., Costantine M.M., Saade G.R., Mucowski S., Hankins G.D., Sciscione A.C. (2010). von Willebrand disease and pregnancy: A practical approach for the diagnosis and treatment. Am. J. Obstet. Gynecol..

[167] Regling K., Kakulavarapu S., Thomas R., Hollon W., Chitlur M.B. (2019). Utility of thromboelastography for the diagnosis of von Willebrand disease. Pediatr. Blood Cancer.

[168] Topf H.-G., Weiss D., Lischetzki G., Strasser E., Rascher W., Rauh M. (2011). Evaluation of a modified thromboelastography assay for the screening of von Willebrand disease. Thromb. Haemost..

[169] Toukh M., Ozelo M., Angelillo-Scherrer A., Othman M. (2013). A novel use of thromboelastography in type 2B von Willebrand disease. Int. J. Lab. Hematol..

[170] Guzman-Reyes S., Osborne C., Pivalizza E.G. (2012). Thrombelastography for perioperative monitoring in patients with von Willebrand disease. J. Clin. Anesth..

[171] Schmidt D.E., Majeed A., Bruzelius M., Odeberg J., Holmström M., Ågren A. (2017). A prospective diagnostic accuracy study evaluating rotational thromboelastometry and thromboelastography in 100 patients with von Willebrand disease. Haemophilia.

[172] Boyd E.Z., Riha K., Escobar M.A., Pivalizza E.G. (2011). Thrombelastograph platelet mapping in a patient with von Willebrand disease who was treated with Humate-P. J. Clin. Anesth..

[173] Topf H.-G., Strasser E.R., Breuer G., Rascher W., Rauh M., Fahlbusch F.B. (2019). Closing the gap–detection of clinically relevant von Willebrand disease in emergency settings through an improved algorithm based on rotational Thromboelastometry. BMC Anesthesiol..

[174] Chi C., Lee C.A., Shiltagh N., Khan A., Pollard D., Kadir R.A. (2008). Pregnancy in carriers of haemophilia. Haemophilia.

[175] Plug I., Mauser-Bunschoten E.P., Bröcker A.H., van Amstel H.K.P., van der Bom J.G., van Diemen-Homan J.E., Willemse J., Rosendaal F.R. (2006). Bleeding in carriers of hemophilia. Blood.

[176] Seligsohn U. (1978). High gene frequency of factor XI (PTA) deficiency in Ashkenazi Jews. Blood.

[177] Martin-Salces M., Jimenez-Yuste V., Alvarez M.T., Quintana M., Hernandez-Navarro F. (2010). factor XI deficiency: Review and management in pregnant women. Clin. Appl. Thromb. Hemost..

[178] Davies J.R., Fernando R., Hallworth S.P. (2007). Hemostatic function in healthy pregnant and preeclamptic women: An assessment using the platelet function analyzer (PFA-100®) and thromboelastograph®. Anesth. Analg..

[179] Paniccia R., Priora R., Liotta A.A., Abbate R. (2015). Platelet function tests: A comparative review. Vasc Health Risk Manag..

[180] Pettersen A.A., Arnesen H., Seljeflot I. (2015). A brief review on high on-aspirin residual platelet reactivity. Vascul. Pharmacol..

[181] Paul B.Z.S., Jin J., Kunapuli S.P. (1999). Molecular mechanism of thromboxane A(2)-induced platelet aggregation. Essential role for p2t(ac) and alpha(2a) receptors. J. Biol. Chem..

[182] Peng H.T., Nascimento B., Beckett A. (2018). Thromboelastography and Thromboelastometry in Assessment of Fibrinogen Deficiency and Prediction for Transfusion Requirement: A Descriptive Review. Biomed Res. Int..

[183] Bande B.D., Bande S.B., Mohite S. (2014). The hypercoagulable states in anaesthesia and critical care. Indian J. Anaesth..

[184] Bell S.F., Collis R.E., Pallmann P., Bailey C., James K., John M., Kelly K., Kitchen T., Scarr C., Watkins A. (2021). Reduction in massive postpartum haemorrhage and red blood cell transfusion during a national quality improvement project, Obstetric Bleeding Strategy for Wales, OBS Cymru: An observational study. BMC Pregnancy Childbirth.

